# Impact of diet in shaping gut virome of grain-fed and grass-fed beef cattle revealed by a comparative metagenomic study

**DOI:** 10.1186/s40168-025-02163-1

**Published:** 2025-08-23

**Authors:** Yujie Zhang, Yen-Te Liao, Fang Liu, Robert W. Li, Vivian C. H. Wu

**Affiliations:** 1 Produce Safety and Microbiology Research Unit, Department of Agriculture, Agricultural Research Service, Western Regional Research Center, Albany, CA 94710 USA; 2 Animal Parasitic Diseases Laboratory, Department of Agriculture, Agricultural Research Service, Beltsville Area Research Center, Beltsville, MD 20705 USA

**Keywords:** Gastrointestinal virome, Grass-fed beef cattle, Grain-fed beef cattle, Post-weaning weight

## Abstract

**Background:**

In the United States beef industry, grain-feeding and grass-feeding are the two most common types of cattle feeding. Different feeding methods are likely to affect gut microbiota compositions and subsequently change microbial adaptation and cattle metabolism. However, there is limited information regarding the impact of diet on cattle gastrointestinal virome. This study examined the composition of fecal virome from grain-fed and grass-fed beef cattle and identified unique virome features to understand the relationship between these two feeding types.

**Results:**

Six grain-fed and six grass-fed Angus beef cattle were weighed, and their fecal samples were collected for further viral metagenomic sequencing. The difference in animal growth revealed a significantly higher post-weaning weight in grain-fed cattle than in grass-fed cattle after day 56. Furthermore, the analysis of the fecal viral population showed that approximately 795 and 1266 predicted viral sequences were obtained in the grain-fed and grass-fed samples, respectively. Among those, 54.3% of the grain-fed and 26.3% of the grass-fed viral sequences were identified as known viruses. The taxonomic classification showed that viruses belonging to the order *Caudovirales*, mostly bacteriophages, dominated the cattle virome in both sample groups, followed by the order *Cremeviriles* and *Petitvirales*. At the family level, 13 and 16 different viral families were detected in the grain and grass-fed groups, respectively. The comparison of virome features from the two groups indicated that the viral population from the kingdom *Bamfordvirae* had a significantly higher abundance in the grain-fed group than in the grass-fed cattle virome. In contrast, the kingdom *Heunggongvirae* had a significantly higher abundance in the grass-fed group than in the grain-fed cattle virome. Moreover, the viruses, belonging to the order *Caudovirales* and the family *Podoviridae*, had significantly higher abundances in the grass-fed virome than in the grain-fed virome.

**Conclusions:**

The findings indicate the influence of animal feeds on the changes in gastrointestinal viral compositions and their potential association with cattle weight gain. The current outcome can contribute to further understanding of phage-bacterial interactions and their underlying mechanisms in regulating the animal host’s metabolism and feed efficiency.

Video Abstract

**Supplementary Information:**

The online version contains supplementary material available at 10.1186/s40168-025-02163-1.

## Background

The USA has the world’s largest cattle-feed industry and beef production to provide high-quality products for domestic and export needs. In general, grass-feeding and grain-feeding are the most common types of cattle feeding in the USA. There has been continuing debate on the advantages of grain-fed cattle and grass-fed cattle [1]. The diet of grain-fed cattle has higher non-fibrous carbohydrates (NFC) and lower neutral detergent fiber (NDF) than the grass-fed diet; these differences in diet structures subsequently affect metabolic regulation, such as the energy production pathway of fatty acids degradation, and result in the distinct nutrient utilization efficiency in beef cattle [2, 3]. For example, the growth rate of grain-fed cattle reaching market weight is faster than that of grass-fed cattle due to the higher feed efficiency [2]. However, the beef industry has claimed that grass-fed beef is rich in certain fatty acids like omega-3 fatty acids (ω−3), omega-6 (ω−6) fatty acids, and a newly discovered fatty acid, conjugated linoleic acid (CLA), which are of significant health benefits [4, 5]. CLA is a product that starts off in the diet as a plant-based fat. After being consumed by the ruminant animal, the unsaturated fatty acids are converted to saturated fats by the rumen bacteria [6, 7]. Thus, these bacteria are mainly responsible for the biohydrogenation of unsaturated fatty acids in the rumen. In addition, the metabolic activity of gastrointestinal microorganisms is vital in providing microbial proteins through digesting nitrogen-containing compounds and influencing animal growth and milk synthesis [8]. These findings demonstrate that gastrointestinal microbiota composition plays a significant role in the animal host’s physiological and production traits.

The microbial community of different ruminant species can co-evolve with their animal hosts and is associated with energy generation from the animal’s low-quality, fiber-rich diets. Flint et al. reported that gut microbes in ruminants, like cattle, contained high densities of bacteria, including polysaccharide-degrading strains, in primary gut locations of microbial breakdown of dietary polysaccharides. These microbial fermentation products, such as short-chain fatty acids, provided energy and nutrients and contributed to about 70% of the animal’s energy intake [9]. Henderson et al. concluded that the rumen microbial ecosystem predominantly contained a core community of an estimated 19 bacterial phyla, such as *Firmicutes* and *Bacteroidetes*, contributing to over 90% of the ruminant gastrointestinal (GI) samples [10, 11]. Compared to beef cattle fed with different diets, Li et al. detected 342 genes with distinct expression levels between ruminal wall samples of grass-fed and grain-fed Angus cattle [12]. Their results showed that 78% of these genes displayed significantly high expression levels in grass-fed steers, with the majority associated with cell development and biosynthesis. Additionally, the authors revealed trait differences between the grass-fed and grain-fed cattle: grass-fed beef had higher concentrations of beta-carotene and glutathione and less total fat than grain-fed beef. Their findings suggested that different dietary conditions contributed to various rumen functions of feed digestion and nutritional absorption via enzymatic activities and metabolic pathways, ultimately influencing the production traits of grass-fed and grain-fed cattle.

In addition to the GI bacteriome, there is an increasing focus on the gut virome of mammals, facilitating a snapshot of the GI virome [13–15]. These articles indicated that bacteriophage (or phage)—the primary component of the GI virome—plays a vital role in modulating the complex gut microbiome. It is well known that bacteria and phages co-exist and co-evolve in the same ecological niche. The changes in the bacterial population caused by viral infection can modify microbial processes in an ecosystem and further result in metabolic reprogramming [16, 17]. A previous study revealed that metabolic functionalities of the viruses associated with microbial ecosystems resulted in significant changes in bacterial metabolism, including relaxing metabolic bottlenecks, complementing microbe-microbe interactions, increasing nutrient-utilizing efficiency, and providing energy to animals [18]. However, the GI virome research on the interaction and regulation between phages and bacteria in farm animals, such as cows, sheep, and goats, is quite limited, with few recent studies published [19, 20]. Most importantly, there is a lack of comprehensive virome studies on the viral population of the GI tract within beef cattle and their association with feeding types. The only study we found utilized a metagenomic approach to examine bovine rumen virome and focused on characterizing the virome composition and variation among cattle fed with different diets [17]. Their study reported that rumen viruses had various responses to different dietary treatments and further impacted the microbial and host metabolisms through the auxiliary metabolic genes (AMG) carried by viruses. Therefore, this study aimed to identify unique virome features related to grain-fed and grass-fed cattle feeding methods via viral metagenomic sequencing. The findings in this study will provide genomic evidence of the diet-mediated variation in rumen gut virome and establish a foundation for future hypotheses regarding the important role of gut virome in the rumen phenotypes and production traits.

## Methods

### Animals and sample collection

Angus steers used in this study were strictly handled according to the animal use protocol approved by the Beltsville Area Animal Care and Use Committee and the Institutional Animal Care and Use Committee at the University of Maryland (UMCP-IACUC Protocol #R-11–72). Twelve Angus steers came from the Wye Angus beef cattle herd maintained by the University of Maryland, located in Queenstown, Maryland, USA, at 38.9907° N, 76.1577° W. Six of them were raised under the free-range grass-fed regime. These animals had free access to grazing alfalfa and/or alfalfa baleage during the cold season. No fertilizers, pesticides, or other synthetic chemicals were used for alfalfa production. The cattle were not provided with any animal, agricultural, or industrial by-products or grain. The six grain-fed cattle received a finishing diet containing silage corn, shelled corn, soybeans, and trace minerals. The metadata (see Table S1), including the date of birth, birth weight, dam, sire, pedigree information, and production traits, were carefully recorded by the production crew. Live animal weight was also measured on days 28, 56, 84, 112, 164, and 226 after birth to calculate weight gain. Analysis of covariance (ANCOVA) was used to determine the statistical difference in weight at each time point after weaning between grain-fed and grass-fed cattle, with the setting of different time points as the within-subject factor, feeding type as the between-subject factor, and the weaning weight as the covariate. At approximately seven months of age, fecal matter in the rectum of these cattle was collected by the production crew within a 2-h time frame on the same day. The samples were then snap-frozen in dry ice and stored at − 80 °C until viral particle separation and viral DNA extraction.

### Total viral DNA isolation, library preparation, and metagenomics sequencing

For viral particle enrichment, approximately 15 g of fecal samples were homogenized with 30 ml of PBS for 10 min. The mixture was centrifuged at 4000×*g* for 10 min. The supernatant was transferred to a new tube before passing through 0.45 μm, followed by 0.22 μm filters. After the filtration, the total volume recovered was approximately 10 to 15 ml. The filtrate was then concentrated using 30K/50K Amicon filters (Millipore Sigma, St. Louis, MO, USA) to a final volume of 200 to 400 μl. The concentrate was treated with DNase I for 1 h before incubating at 75 °C for 15 min to inactivate the DNase I. Viral DNA was extracted from 200-μl viral concentrate using an AllPrep PowerViral DNA/RNA (Qiagen, Germantown, MD, USA). The crude viral DNA was then purified using a Qiagen DNeasy PowerClean CleanUp Kit. The quality and concentration of viral DNA were confirmed using Agilent High Sensitivity DNA Kits (Santa Clara, CA, USA). The viral DNA was then amplified using an illustra^™^ Single Cell GenomiPhi^™^ DNA Amplification Kit (MilliporeSigma) following the manufacturer’s instructions. The quality of amplified viral DNA was verified using an Agilent DNA 7500 kit, and the concentration was measured using a Qubit fluorometer. The viral DNA library was constructed using a NEBNext® Ultra™ DNA Library Prep Kit for Illumina (New England Biolabs (Ipswich, MA, USA) and then sequenced using an Illumina HiSeq 2000 sequencer.

### Bioinformatics and data analysis

The raw sequence reads of cattle fecal virome were quality filtered and trimmed using FastQC (version 0.11.9) and Bbmaps (version 38.79), with the average quality setting at Q20 [21, 22]. To determine the quality of viral metagenomic reads, bacterial contamination was assessed by mapping the reads against rRNAs and single-copy bacterial markers via ViromeQC1.0, with the default settings [23]. The qualified reads from six grain-fed cattle and six grass-fed cattle virome were co-assembled using Megahit (version 1.2.9), respectively, with the settings of –k-min 21 –k-max 149 –k-step 24 -m 0.99 –min-contig-len 1000 -t 72 –k-min-1pass [24]. For viral population identification, the co-assembly contigs of each group were filtered with DeepVirFinder 1.0, and the contigs, having *q*-values < 0.01, were predicted as viruses [25]. The rest of the contigs, which were longer than 10 kb, remained unclassified viruses for further analysis. The viral genomes, including identified and unclassified viral sequences, were clustered using CD-HIT (version 4.7, default setting) to reduce redundancy. The resultant viral contigs (referred to as final virome) were subjected to blast against the RefSeq viral database for taxonomy classification (obtained on 01/09/2022; *E*-value threshold of 10^−5^, a bit-score threshold of 50). The relative abundances of viral populations were obtained by mapping Illumina viral reads to contigs using Bbmap v38.79, with the default settings. Principal component analysis (PCA) at the viral family level was further conducted using the FactoMineR package (version 2.7) and ggplot2 package (version 3.4.1). To determine the influences of feeding types on the viral composition, the Linear Discriminant Analysis (LDA) Effect Size (LEfSe) algorithm was used to identify the significantly different taxa of grain-fed and grass-fed cattle virome, with an LDA score of more than 2, based on the relative abundance of viral taxa of two groups [26]. In addition, the final viromes were subjected to annotation using RAST (version 2.0; the domain of virus) and further submitted to KofamKOALA (version 2022–01–03) with default parameters to identify the functional genes.

## Results

### Animal sampling and performance information

**Fig. 1:**
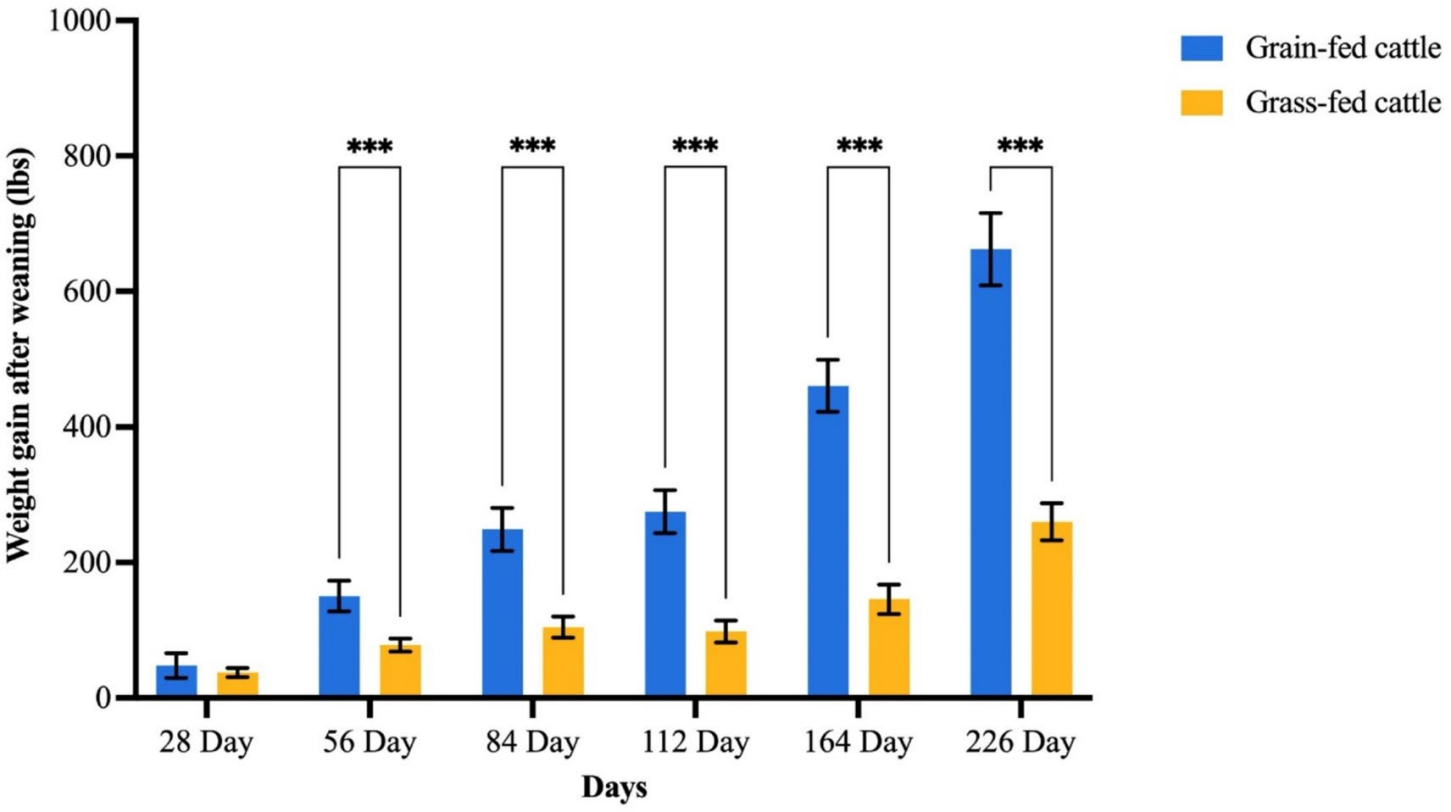
The weight gain of grain-fed and grass-fed beef cattle adjusted for weaning weight. ***p < 0.001

To determine the dietary effects on beef cattle growth, the body weight from two feeding groups was recorded during the production process. While the average birth weight of the calf assigned to the two feeding groups was similar (*p* = 0.45), the weaning weight of grain-fed cattle was significantly lower than that of grass-fed cattle (*p* = 0.02) (Table S2). Therefore, weaning weight was included as the covariate in the ANCOVA to compare the growth difference related to the feeding types. A significant main effect of feeding type (*p* = 0.005) and a significant main effect of different time points (*p* < 0.001) were shown in the analysis (Table S3). Most of all, a significant interaction between feeding type and different time points (*p* < 0.001) was also found, suggesting the growth of grain-fed cattle was significantly different from that of grass-fed cattle (Table S3). To better interpret this interaction, the post-hoc pairwise comparisons between grain-fed and grass-fed groups at each time point were conducted based on the weaning weight. Although the weight gain was not statistically different on day 28 between the two feeding groups (*p* = 0.21), grain-fed cattle showed significant weight gain compared to the grass-fed cattle from day 56 to the last sample collection on day 226 (*p* < 0.001; Fig. 1, Table S4). The results indicated the significant influence of feeding types on the animal’s post-weaning weight.

### Sequencing information

A total of 12 fecal viral samples were sequenced in this study. After the quality control, an average of 58,474,837 reads per sample was obtained, with a range of GC contents from 40.1 to 50.2% (Table 1). The bacterial DNA contamination of each sample was lower than 0.2%, indicating high quality of these viral metagenomic sequencing data for downstream analyses [27]. Further, the six samples from each feeding type were co-assembled and annotated. The results showed that the grain-fed fecal virome contained a total of 50,508 contigs with 196,228 predicted genes, while the grass-fed group had a significantly higher number of 104,679 contigs with 412,861 predicted genes (Table 1).

**Table 1:** Quantitative information and quality control of virome sequencing

Sample	Raw reads	Clean reads	Q20 (%)	Average read length (bp)	GC content (%)	Bacterial contamination (%)	Contigs	N_50_	Prediction genes
GN*_0985	55,704,174	53,626,434	96.27	135.8	41.8	0.013	50,508	2820	196,228
GN_1050	62,386,632	60,225,512	96.54	135.3	42.6	0.007			
GN_1067	52,342,982	51,036,216	97.50	139.4	50.2	0.002			
GN_1106	53,240,764	51,807,434	97.31	138.5	43.9	0.066			
GN_1121	51,854,320	50,432,772	97.26	138.8	44.9	0.024			
GN_1127	54,997,426	53,547,896	97.36	138.1	47.8	0.002			
GS_0989	67,496,920	65,576,710	97.16	136.3	40.6	0.013	104,679	2702	412,861
GS_1041	68,028,458	66.052,304	97.01	135.8	40.9	0.008			
GS_1059	75,855,488	74,072,018	97.65	136.4	42.4	0.020			
GS_1078	72,311,676	70,367,474	97.31	134.8	46.5	0.004			
GS_1103	66,209,480	64,274,970	97.08	136.1	41.2	0.005			
GS_1122	50,141,408	48,255,768	96.24	132.7	40.1	0.019			

*GN refers to grain-fed cattle feces, while GS refers to grass-fed cattle feces

### Fecal viral communities influenced by diets in beef cattle

**Fig. 2:**
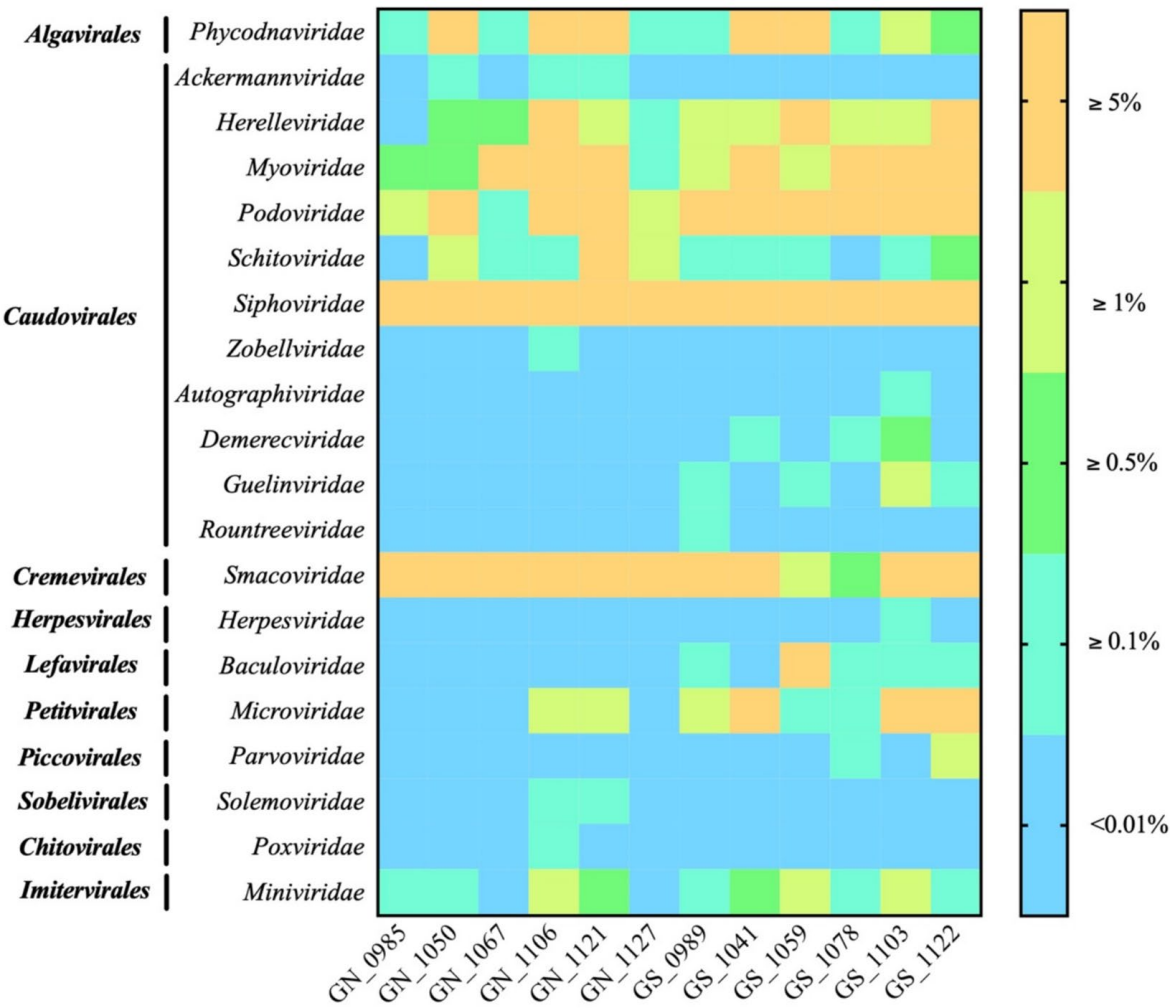
Relative abundance (percentage of raw reads mapped to classified viral contigs) of known viral families identified from the viral metagenome in grain-fed and grass-fed cattle feces using the Refseq database. GN refers to grain-fed cattle feces, while GS refers to grass-fed cattle feces

A total of 795 and 1266 predicted viral sequences (referred to as viral genomes) composed the viral community of grain-fed and grass-fed cattle feces, respectively. Furthermore, 432 (54.3%) and 333 (26.3%) viral sequences from grain-fed and grass-fed samples, respectively, shared high nucleotide similarities with known viral genomes. Among all the known viral genomes, there were 10 viral families under six different viral orders predicted in both sample groups (Fig. 2). Three viral families—*Siphoviridae* (12% in both groups), *Podoviridae* (1.7% and 7.5% in grain-fed and grass-fed groups, respectively)*,* and *Myoviridae* (1.9% in both groups)—had the highest relative abundance under the order *Caudovirales*. The *Smacoviridae* family under *Cremeviriles* (15.2% and 4.2% in grain-fed and grass-fed groups, respectively) and the *Microviridae* family under *Petitvirales* (0.1% and 1.2% in grain-fed and grass-fed groups, respectively) were also shown at a high relative abundance in both groups. In addition, three unique viral families—*Ackermannviridae, Solemoviridae*, and *Zobellviridae—*were only found in the grain-fed fecal virome, with a low relative abundance. On the contrary, there were seven viral families, including *Autographiviridae, Baculoviridae, Demerecviridae, Guelinviridae, Herpesviridae*,* Parvoviridae,* and *Rountreeviridae*, only detected in the grass-fed fecal virome. The top 10 viral families within the two groups were further subjected to the principal component analysis, and ordination plots further revealed a distinct representation of viral communities between grain-fed and grass-fed cattle groups with three associated viral families (*Schitoviridae, Smacoviridae*, *Miniviridae*) along dimensions 1 and 2 (*p* < 0.01) (Fig. S1). Altogether, the results indicated that the difference in viral composition between these two groups was likely due to various dietary treatments, with the grass-fed fecal virome exhibiting a higher viral diversity than the grain-fed fecal virome.

### Differentially abundant taxa and important microbial features of fecal viral communities under different diets

**Fig. 3:**
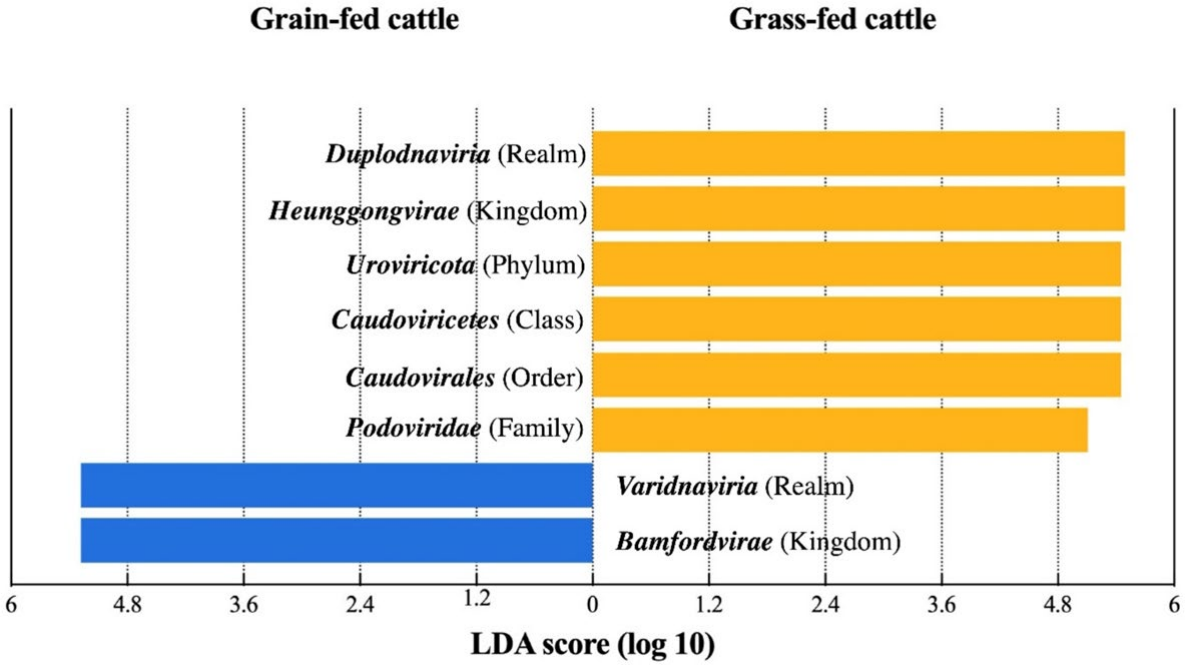
Significantly discriminative taxa with LDA score > 2.0 in fecal viral metagenome between grain-fed and grass-fed beef cattle were determined by the LEfSe algorithm analysis and displayed based on the ICTV taxonomic level

Next, the LEfSe algorithm was used to determine the difference in taxa between the two groups. The results indicated that the viral populations from grass-fed and grain-fed feeding groups were significantly different in the taxonomic level, with an LDA score > 2 (Fig. 3). Specifically, a high abundance of viruses, belonging to the kingdom *Heunggongvirae* of *Duplodnaviria* realm, was shown in the grass-fed fecal virome; however, the viruses in the grain-fed fecal virome were highly abundant in the kingdom *Bamfordvirae* under the realm *Varidnaviria*. Moreover, compared to the grain-fed group, the grass-fed group contained significantly higher abundance of viruses in different taxa, including the phylum *Uroviricota*, the class *Caudoviricetes*, the order *Caudovirales*, and the family *Podoviridae*, with LDA scores of more than 5.

### Virus-encoded functional genes are involved in diverse metabolic pathways

**Fig. 4:**
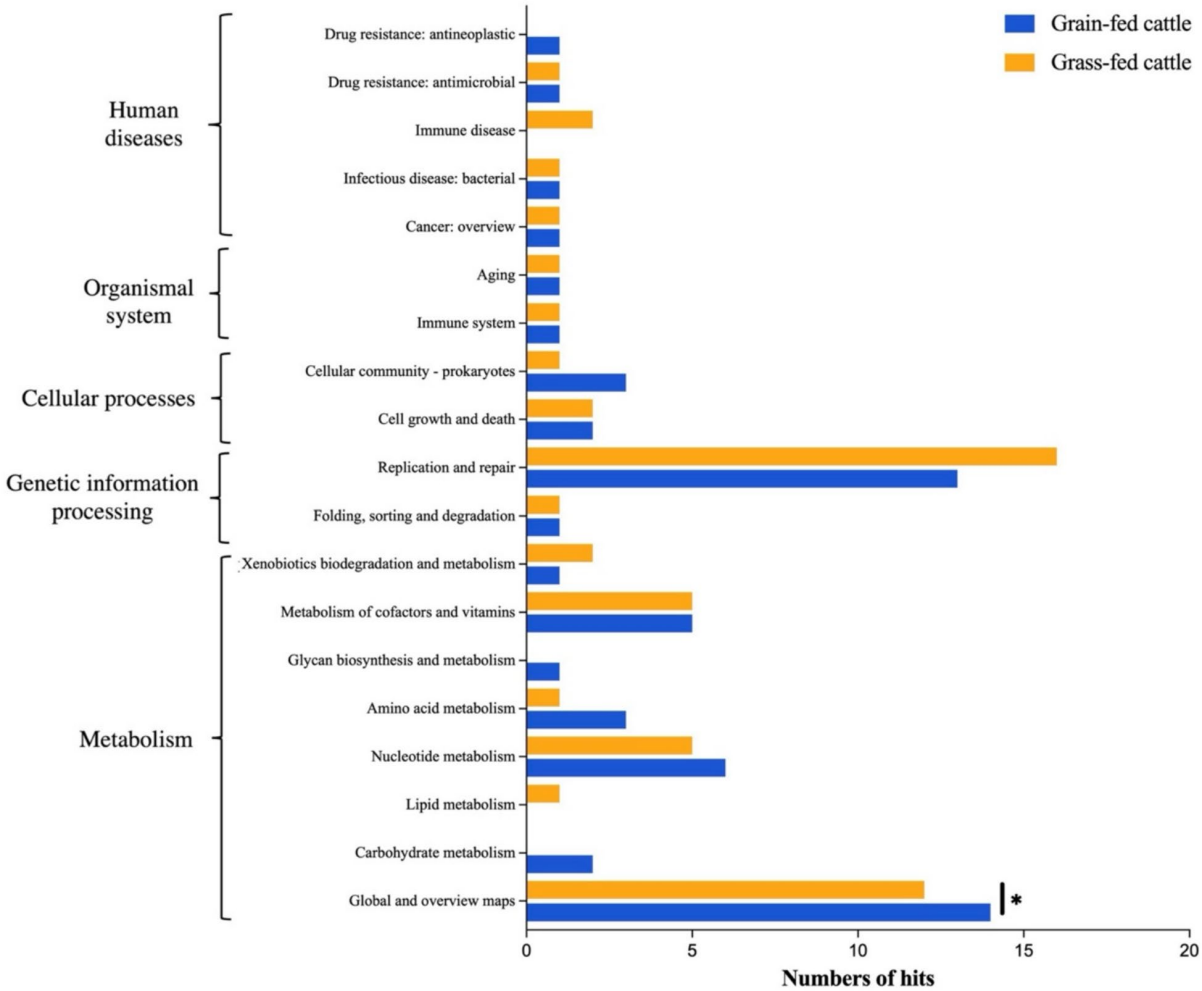
The number of functional genes encoded by viruses from the grain-fed and grass-fed cattle feces based on the annotation using KofamKOALA. *The significance level was calculated by Fisher’s exact test with *p* < 0.05

To explore the contribution of viruses to microbial ecology, the functional genes in the viromes from two feeding groups were identified and compared in Fig. 4. Various functional genes, involved in metabolism, human disease, organismal system, cellular processes, and genetic information processing, were detected from these two types of fecal viromes (Table S5). The functional genes of viruses were primarily related to the “Metabolism” and “Genetic information processing” categories. For example, the genes involved in the pathway of replication and repair, such as DNA replication and homologous recombination, were detected in both groups with the highest hits (Fisher’s exact test, *p* = 0.1648). The genes under the pathway of “global and overview maps” displayed significantly higher numbers in the grain-fed group compared to the grass-fed group (Fisher’s exact test, *p* < 0.05), most of which were related to metabolic pathways and biosynthesis. Both grain-fed and grass-fed fecal viromes also carried unique functional genes belonging to the “Human diseases” and “Metabolism” categories. Specifically, the genes related to immune disease and lipid metabolism were only detected in the grass-fed group; however, those related to antineoplastic drug resistance, glycan biosynthesis and metabolism, and carbohydrate metabolism were only identified in the grain-fed group (Fisher’s exact test, *p* > 0.05).

## Discussion

Recently, many studies have explored the composition and the role of viral populations in different environmental samples and have indicated that the virome drives the diversity and evolution of the microbial population [28, 29]. Gut microbes (including fungi, archaea, bacteria, and viruses) and their interactions with each other play critical roles in host energy acquisition and meat production traits of farm animals [30–32]. Studies have identified viral populations implicated in modulating these complex communities. However, most studies regarding farm animal viromes typically focus on the interaction between viruses and human-related diseases, such as respiratory and gastrointestinal diseases. Thus, our study aimed to investigate viral composition and unique features of viromes obtained from the grain-fed and grass-fed beef production systems to understand the potential effects of feeding types on the gut viromes.

Phages, the viruses that infect bacteria, are the major members of the mammalian gastrointestinal virome [14]. It has been reported that the gut phageome mainly interacts with the dominant ruminant bacteria *Firmicutes* and *Bacteroidetes*, altering the ecology and evolution of microbial communities [33, 34]. Consistent with the viral profile from different mammalian GIT samples, *Caudovirales* phages dominate both grain-fed and grass-fed cattle fecal viromes, likely co-evolving with diverse bacterial hosts and shaping the beef cattle gut microbiome [35–38]. However, the detailed interactions between phages and bacteria in the gut microbiota are poorly understood. Remarkably, only one review article on gut microbiota suggested that phages displayed different dynamics to interact with their bacterial hosts in the gut environment, subsequently altering the gut microbiota. In general, the mammalian GI tract is a heterogeneous ecosystem, with spatial distribution amongst different phage populations depending on diverse factors such as gut location and bacterial concentration [36]. Although the information regarding the cattle gut phagenome remains scarce, one study conducted by Rincón reported that phages in the *Myoviridae* family were the most abundant viral family in the large intestine of cattle [39]. In the current study, *Siphoviridae* phages have the highest relative abundance in both grain-fed and grass-fed cattle fecal samples compared to other phage families. Therefore, the results of this study, with the fecal virome profile of beef cattle, elucidate the gap regarding the cattle phage population from different GI sites. These findings also provide the foundation for exploring the phage-bacteria interactions and their roles in the gut microbial ecology and evolution.

Generally, the virome in gut microbiota is vastly varied among different animal species. Within each animal species, multiple viruses are intertwined with one another and vital to the gut environment. They are unique to the individual animal due to their diet, farming environment, and maternal lineages. Particularly, animal feeds are found to widely influence gut microbiota, including the virome [40]. In our study, the grass-fed cattle had a higher viral diversity than the grain-fed groups. The current result was consistent with previous studies that found a higher microbial diversity in feces of grass-fed cattle than in grain-fed cattle [41, 42]. As the present study is the first to report on the cattle virome in relation to different feeding types, the higher abundance of *Podoviridae *in the grass-fed group could serve as a valuable factor associated with the potential effects of diet changes on the gut phageome that further affect the microbial community. For example, *Podoviridae* phages were frequently isolated from cattle feces with lytic activity against *Escherichia coli*, *E. coli* O157:H7 in particular [43, 44]. The study regarding comparison of the gut microbiota also indicated that the prevalence of *E. coli* O157:H7 in grain-fed cattle was higher than in the grass-fed groups, posing a potential public health issue [45]. The higher abundance of *Podoviridae* might explain the low prevalence of *E. coli* in the grass-fed cattle group, and could serve as a potential indicator for quality control and food safety in the beef industry.

Additionally, feed efficiency is an important measure in beef cattle production that can be affected by several factors, such as nutrition, metabolism, and behavior, depending on numerous biochemical pathways [46]. Weight, a parameter of residual feed intake (RFI) used to measure feed efficiency, best explains the metabolic variation between two feeding types. This study demonstrated that the body weight of grain-fed cattle was significantly higher than that of grass-fed cattle after day 56, suggesting a higher RFI of the grain-fed cattle [47]. Consistent with our body weight results, the grain-fed cattle virome had a significantly higher abundance of the microorganisms belonging to the kingdom *Bamfordvirae*. Under *Bamfordvirae*, three viral families identified in the grain-fed group, including *Mimiviridae*, *Phycodnaviridae*, and *Poxviridae, *were classified in the phylum *Nucleocytoviricota* (NCV). The *Nucleocytoviricota *phylum is notable for containinggiant viruses. The complexity of NCVs regarding host range, lifestyles, and metabolic capabilities has not been well studied. Previous studies reported that NCVs could infect diverse eukaryotic lineages and further regulate microbial metabolism via various processes, such as cell lysis, horizontal gene transfer, metabolic reprogramming, and lysogenization [48–50]. Another study indicated that diverse metabolic genes, such as those involved in fermentation and diverse substrate transport processes, were detected in NCV genomes [49]. These genes represent another form of host metabolism manipulation by integrating into the host genome and expanding the catalytic capabilities of eukaryotic cells, especially in harsh environments [40, 48, 51, 52]. In addition, NVCs that display lytic activity could lyse host cells and release diverse molecules into the surrounding environment, increasing the nutrition components [53]. It is not clear how NCVs influence rumen eukaryote populations; however, the eukaryotic community, such as fungi and protozoa, is responsible for many functions in the rumen and is beneficial to the host animals [54–58]. For example, rumen fungi play a critical role in fiber degradation via penetrating the cuticle and cell wall of lignified material [55]. Other studies demonstrated that protozoa could engulf starch and attach amylolytic bacteria to further regulate starch fermentation rate in the rumen [57, 58]. Overall, differences in the ruminal eukaryotes, modulated by the diet types, could contribute to variations in animal feed efficiency [59]. Due to complicated interactions among rumen microflora, there is still no direct evidence showing that giant viruses are contributing to the body weight of grain-fed cattle. However, the varying viral communities from two types of feeding groups in this study appear to have consequences for microbial metabolism that are largely congruent with the current paradigm established in the rumen eukaryotic ecosystem. Thus, our study provides the fundamentals for understanding the effect of diet on the changes in gut virome and their potential influence on rumen metabolism.

Phages play significant roles in the ecology of the natural environment, human, and animal microbiome through their interactions with bacterial hosts [17, 60, 61]. Phage-mediated horizontal gene transfer, particularly auxiliary metabolic genes (AMGs), is one of the primary factors altering animal metabolism and has been confirmed in many studies [60, 62, 63]. For example, Chen et al. detected a *pmoC* gene from the phages isolated from lake water that encoded a subunit of a particulate methane monooxygenase, a predominant methane oxidation catalyst in nature [60]. Zheng et al. reported that viruses isolated from soil harbored a high relative abundance of AMGs linked to pesticide degradation and metabolism [64]. In the current study, comparing AMGs carried by viruses between grain-fed and grass-fed viromes allowed us to focus on the functional genes related to prokaryotic cellular processes. Interestingly, unlike the grass-fed cattle fecal viromes, the viromes in the grain-fed cattle feces harbored the genes related to quorum sensing, biofilm formation of *Vibrio cholerae*, and biofilm formation of *Escherichia coli*. The presence of those virulence-related genes contributing to the fitness of bacterial hosts may be associated with a high abundance of bacterial pathogens, such as *Escherichia coli*, in grain-fed cattle [45]. Virulence genes and those associated with human disease pathways, such as those related to antimicrobial and antineoplastic drug resistance, pose a potential risk to the animal host and human health in general. These genes in the cattle virome caught our attention for future investigation regarding the correlation among gut virome, animal, and human health. In addition, the genes related to lipid metabolism in the grass-fed virome and those involved in carbohydrate metabolism in the grain-fed group further justified the potential benefits of these two groups regarding fatty acid synthesis and beef production, respectively [65]. Our findings serve as a backbone for understanding the correlation between animal hosts’ phenotypes and the functional genes of viruses. Further studies are needed regarding the metabolic regulation mediated by phage-bacterial interactions as well as their contribution to the growth of animal hosts.

In the current study, approximately 46% (in the grain-fed group) and 74% (in the grass-fed group) of viral sequences were uncovered and shared no similarity with known viruses, as the viral dark matter of the gut virome. A previous study reported similar results, indicating that viral dark matter comprised 40 to 90% of the sequences based on the sample types [66]. For example, the gut virome isolated from bovine rumen fluid contained a lot of unknown viruses (∼ 78%) based on the sequences matched to the previously published data [67]. Identifying viral genomes from metagenomics is the fundamental step for future studies to investigate the role of viromes in the gut microbiome; therefore, database-based and alignment-free methods have been designed and commonly used for viral detection. In contrast to the traditional alignment-based methods, the DeepVirFinder tool outperformed the rest at all sequence lengths with high accuracy (based on the DeepVirFinder results). Both tools, Virsorter and DeepVirFinder, were used in this study to identify the taxonomy of the viral sequences after a blastn search of the output data [25, 68]. The current results showed that a total of 135 contigs belonging to viral sequences were classified via the Virsorter analysis, but 217 contigs were identified as viral sequences by the DeepVirFinder (data not shown). The inconsistent results obtained from two different tools highlight the need to develop new bioinformatics approaches to facilitate research on the viral dark matter within the gut virome. Nevertheless, the current findings reveal different compositions of the cattle gut virome and show significant differences in viral features between grain-fed and grass-fed beef cattle.

## Conclusions

In conclusion, the present study demonstrated the influence of different animal feeding types in the composition of the gastrointestinal virome of beef cattle, particularly DNA viruses, and its potential association with cattle production traits. The unique virome features related to the two feeding methods also contributed to the investigation of its potential interactions with other gut microorganisms and the subsequent changes in the animal host’s metabolism. Future research is critical to focus on the detailed functional analysis of the gut virome and its contribution to the physiological and production traits of animal hosts via metabolic regulation.

## Supplementary Information

Additional file 1: Table S1. The production traits of grain-fed and grass-fed beef cattle. Table S2. Independent t-test of birthweight, weaning height, and weaning weight between grain-fed and grass-fed cattle. Table S3. Analysis of covariance (ANCOVA) of post-weaning weight between grain-fed and grass-fed cattle. Table S4. Post-hoc pairwise comparisons of weight gain between grain-fed and grass-fed cattle since weaning. Table S5. The KEGG pathway of functional genes encoded by viruses from the grain-fed and grass-fed cattle feces based on the annotation using KofamKOALA.

Additional file 2: Figure S1. Principal component analysis (PCA) plots between grain-fed and grass-fed beef cattle fecal virome with 95% confidence ellipses. Arrows display the directions and relative importance of three viral families associated with the two dimensions as vectors.

## Data Availability

The datasets of cattle viral metagenomes generated during the current study are available in the NCBI Sequence Read Archive (SRA) with the accession number PRJNA845803 [https://www.ncbi.nlm.nih.gov/bioproject/PRJNA845803/].
