# Supplementary figures and images for: Impact of diet in shaping gut virome of grain-fed and grass-fed beef cattle revealed by a comparative metagenomic study
Source: Microbiome. 2025 Aug 23;13:190. doi: 10.1186/s40168-025-02163-1 (PMC12374297; doi:10.1186/s40168-025-02163-1)

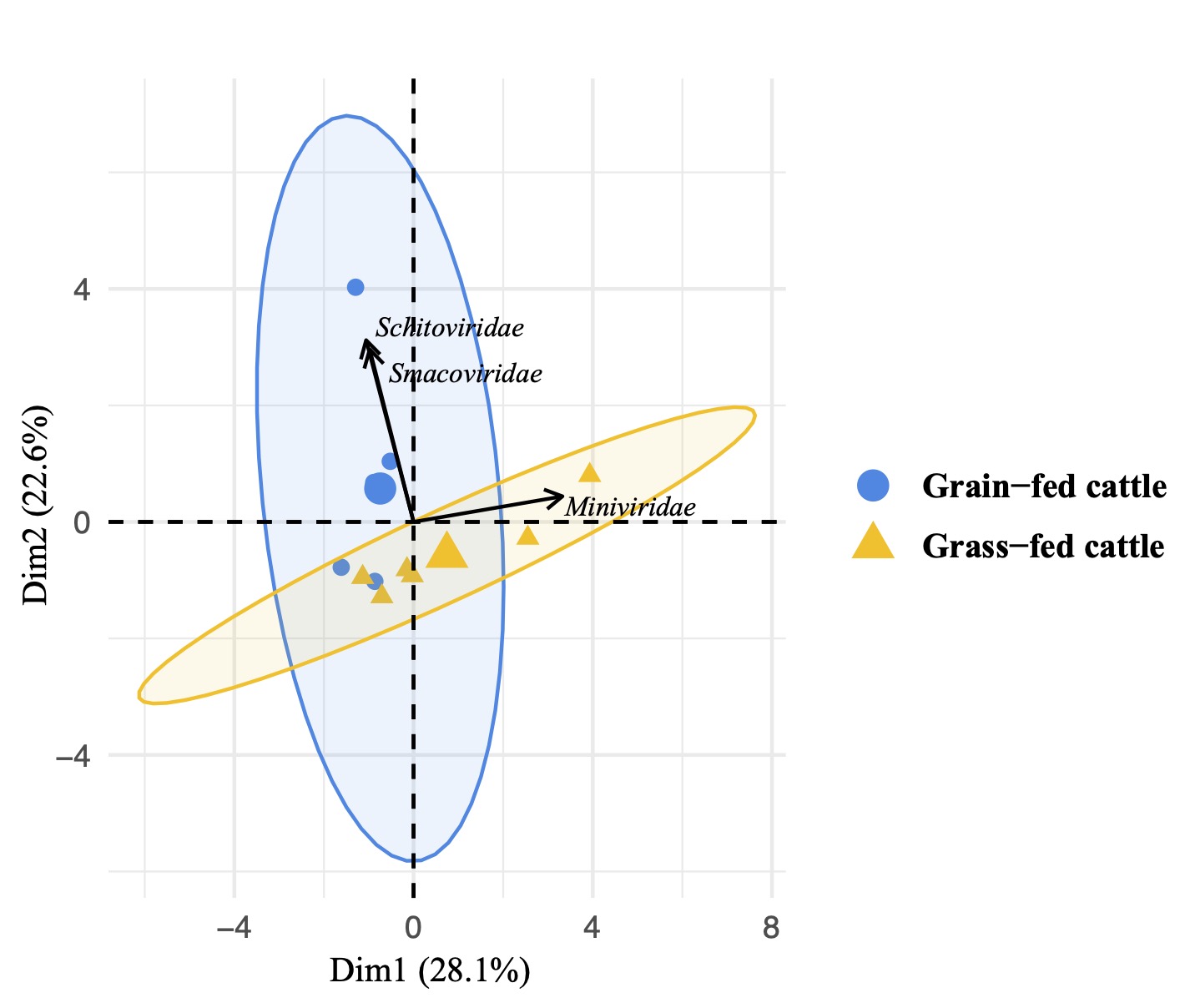

Supplement: Supplementary file 3 — Additional file 2: Figure S1. Principal component analysis (PCA) plots between grain-fed and grass-fed beef cattle fecal virome with 95% confidence ellipses. Arrows display the directions and relative importance of three viral families associated with the two dimensions as vectors. [file 40168_2025_2163_MOESM2_ESM.jpg]
